# Assessing the health-related quality of life of nurses: A study in hedi chacker hospital

**DOI:** 10.1192/j.eurpsy.2021.1066

**Published:** 2021-08-13

**Authors:** A. Abbes, A. Kchaou, R. Masmoudi, A. Hrairi, K. Jmal Hammami, M. Larbi Masmoudi, J. Masmoudi, M. Hajjaji

**Affiliations:** 1 The Department Of Occupational Medicine And Work-related Pathology, University hospital Hedi Chaker Sfax, SFAX, Tunisia; 2 Psychiatry A, hedi chaker hospital, Sfax, Tunisia; 3 Department Of Occupational Medicine, HEDI CHAKER hospital, SFAX, Tunisia; 4 Psychiatrie “a” Department, Hedi Chaker Hospital University -Sfax - Tunisia, sfax, Tunisia; 5 Occupational Medecine, university hospital Hédi Chaker-Sfax, Tunisia, Sfax, Tunisia

## Abstract

**Introduction:**

Physical and psychological health, social relationships and professional environment determine the quality of life of nurses.

**Objectives:**

This study aims to evaluate the quality of life of Hospital nurses and to identify the factors that influenced this assessment.

**Methods:**

We conducted a cross-sectional study concerning nurses who answered a questionnaire developed and structured in order to assess the quality of life and with the appliance of the World Health Organization Quality of Life Instrument (WHOQoL-BREF). The statistical processing was done with IBM SPSS Statistics for Windows, Version 21.0.

**Results:**

Most of the nurses were women (78.30%), married (81.70%) and with a specialization degree (65 %). The mean age of the study population was 50.20 years (±7.20 years). Most of them considered their quality of life good or very good (46.7%) and were satisfied or very satisfied with their health (48.4%). There is a significant association between quality of life and satisfaction with life (p=0.000). The average score of Physical health was 55.76(±13.89). The average scale of Psychological health was 61.45 (±15.14). A significant correlation was observed between lack of antecedents and a better physical health (p=0.000).The psychological health was correlated with age (p=0.000) and social relationships (p=0.000).

**Conclusions:**

Studying the quality of life of nurses is particularly important, since it can lead to the development of a policy for improving the working conditions in the public sector.

**Conflict of interest:**

No significant relationships.

